# Determinants of satisfaction with health care provider interactions at health centres in central Ethiopia: a cross sectional study

**DOI:** 10.1186/1472-6963-10-78

**Published:** 2010-03-24

**Authors:** Zewdie Birhanu, Tsion Assefa, Mirkuzie Woldie, Sudhakar Morankar

**Affiliations:** 1 Jimma University, Public Health Faculty, Department of Health Education and Behavioural Sciences, Jimma, Ethiopia; 2 Jimma University, Public Health Faculty, Department of Health Services Management, Jimma, Ethiopia

## Abstract

**Background:**

In primary health care, provider-patient interaction is fundamental platform and critically affects service delivery. Nevertheless, it is often ignored in medical research and practice and it is infrequently subjected to scientific inquiry, particularly in Ethiopia. This study aimed to assess patient satisfaction with health care provider interactions and its influencing factors among out-patients at health centers in West Shoa, Central Ethiopia.

**Methods:**

A cross sectional facility based study was conducted on 768 out-patients of six health centers in West Shoa Zone, Central Ethiopia. The total sample size was allocated to each of the six health centers based on patient flow during the ten days prior to the start of data collection. Pre-tested instruments were used for data collection and the data were analyzed using SPSS version 16.0 statistical software. Factor score was computed for the items identified to represent the satisfaction scale by varimax rotation method. Using this regression factor score, multivariate linear regression analysis was performed and the effect of independent variables on the regression factor score was quantified.

**Results:**

Seventy three percent of the respondents perceived that provider's empathy was good and 35% complained that providers were not technically competent enough. In addition, 82% of the respondents rated non-verbal communication by the providers to be good, very good or excellent on a five-point ordinal scale. Regardless of the process, only 34.1% of the patients implied that the consultations made a difference in understanding their illness and coping with it. Generally speaking, 62.6% of the patients reported that they have been satisfied with their visit. Perceived empathy, perceived technical competency, non-verbal communication, patient enablement, being told the name of once illness, type and frequency of visit, knowing the providers and educational status were main independent predictors of patient satisfaction in this study. Furthermore, very good empathy (Beta = -4.323), fair non-verbal communication (Beta = -0.188), fewer expectations met (Beta = -0.169) and disagreement to technical competency (Beta = -0.156) had greater negative influence on patient satisfaction. On the other hand, excellent non-verbal communication (Beta = 0.114) and being told the name of once illness (0.109) had pronounced positive influence on patient satisfaction.

**Conclusion:**

The present study showed that interpersonal processes including perceived empathy, perceived technical competency, non-verbal communication and patient enablement significantly influence patient satisfaction. Therefore, health care providers should work towards improving the communication skill of their professionals along with having technically competent workers which could possibly affect the perception of the patient about all of the variables identified as independent predictors of patient satisfaction in this study.

## Background

A wealth of literature reflects the progress made in developing tools to monitor and improve the quality of health care. In developing countries, however, interest in the issue has been surprisingly low until recently. This is so, in spite of overwhelming published and anecdotal evidence of low quality of care in these countries [1].

In Ethiopia, health services are limited and of poor quality [2] and the country has extremely poor health status relative to other low-income countries. To solve this problem, the government has focused on improving the organization and quality of health services delivered to the population. This is because improving the poor quality of care delivered to patients is one of the strategies to reduce the burden of communicable diseases and plays a significant role in attaining the Millennium Development Goals (MDGs). This intention of the government was reflected in the 1993 health policy and the health sector development plans of the country. In such efforts towards improving quality of health care, patient satisfaction is integral component of health services provided to the population [3-11].

It is generally agreed that satisfaction data play significant role in the strategy and tactics health care providers use in delivering services for clients. In addition, measurement of patient satisfaction is increasingly playing important role in the growing push towards accountability among health care providers. It is also viewed as an established indicator of quality of care despite it was overshadowed by measures of organizational aspects in the quality of health care equation [12,13].

Empathy, which is a core component of consultation, is often seen as crucial to the effective achievement of patient satisfaction in that it encapsulates sensitivity to both the informational and emotional aspects of communication [14]. Even though, many standards and codes of practice refer to the importance of empathy in medical consultation, it is an aspect of practice which is too often overlooked [15,16]. Non-verbal cues and languages convey information which words alone often do not. Providers who appear fully attentive, avoid distractions, smile, and sit on the same level as the patient all convey an important message of caring, listening, and empathy [2]. Besides, studies have documented that patient enablement also plays a significant role in patients' overall satisfaction [17,18]. It is also clear from the literature that although system aspects such as cost, access, availability and waiting time are related to patient satisfaction, they have always been identified as being less important than the human aspect of medical care. However, system factors asymmetrically guzzle much of the research topics regardless of their little contribution [4,5]. This is particularly true in the case of developing countries such as Ethiopia where much of the scientific inquiries in the areas of patient satisfaction exclusively focus on organizational aspects [18-20].

Furthermore, established evidences depicted that even though technical aspect of care has its impact on satisfaction, it is through interpersonal communication that the technology of western world reaches the patients and curing occurs. In addition, it is recognized more than ever that the quality of health care for the 21^st ^century is built on the premise that optimal health care can best be achieved in the context of long term relationship between providers and patients [1,6]. However, the issue of patient-provider interaction and its effect on the quality of care rendered at health care facilities is often ignored in medical researches and rarely subjected to scientific inquiry. Therefore, this study aimed to assess patient satisfaction with health care provider interactions and its influencing factors among out-patients of six health centres in West Shoa, Central Ethiopia. Hence, the research question of this study was: *"what are the major determinants of patient satisfaction with their health care provider interaction in resource poor settings such as public health centres in Central Ethiopia?"*

## Methods

### Study area

A cross sectional study was conducted from 29^th ^December - 21^st ^January, 2009 in West Shoa, Central Ethiopia. West Shoa is one of the 17 zones of the Oromia Regional State in Ethiopia and it comprises 21 districts. The zone has an estimated total population of 2,072, 485 of whom 1,037,159 are females.

All the health centres included in the study are government run facilities. The composition of health professionals in these facilities includes health officers, nurses, pharmacy technicians, laboratory technicians and environmental health technicians. However, Shortage of staff in the health system of Ethiopia has always been critical. For instance, health worker to population ratios are 3 to 4 times lower than even the East African standards. Hence, all the public health facilities included in this study function in this context which has clear implication on the quality of care delivered to their clients [2].

### Participants

The study population was included patients who visited the adult medical out-patient departments (OPDs) at six health centers in West Shoa during the study period. A patient was included in the study if he/she is 15 years of age or older. The sample size was calculated assuming p, proportion of patients satisfied with provider interaction, to be 50%. This has been preferred for the sample size determination due to lack of similar studies in Ethiopia. Other assumptions made during the sample size calculation were 5% marginal error (d) and confidence interval of 95%. Based on these assumptions, the sample size was calculated as follows:

This yields a sample size of 384. However, this was multiplied by a factor of 2 to correct the design effect of cluster sampling and the final sample size was **768**.

### Sampling technique

In West Shoa, there are twenty government run health centers. Of these eighteen health centers are located in rural districts while the remaining two belong to urban districts. In this study, one urban and five rural health centers were randomly selected to be included in the study. The total sample size was proportionally allocated to the six health centers based on the number of out-patients 15 years or older served at the adult medical OPDs during the ten days prior to the start of data collection. Finally, consecutive patients who full fill the inclusion criterion (age 15 years or older) were included in the study until the allocated size was obtained in each of the six health centers.

### Measurements

The following instruments were adapted from similar studies:

#### Consultation and Relational Empathy (CARE)

The Consultation and Relational Empathy tool measures the patient's perception of the provider's empathy during the clinical encounter. Within the CARE tool patients were asked 10 questions to rate different aspects of empathy. Each question was scored on an ordinal scale from 'poor' to 'excellent. All ten items taken together yield a maximum score of 50 and a minimum of 10. Higher score on each item indicates higher level of empathy [21,22]. These 10 items were based on the following questions: Thinking about your today's visit, how was your provider at making you feel at ease, letting you tell your story, really listening, being interested in you as a whole person, fully understanding your concerns, being caring and compassionate, being positive, explaining things clearly, helping you to take control, and involving you in decision about treatment plan. The reliability coefficient (Cronbach's alpha) of the empathy scale was 0.964 indicating that the scale was internally consistent. To examine the underlying factors (components) of the empathy scale, an exploratory factor analysis was conducted and produced one meaningful factor with eigenvalue greater than one. This factor accounted for 75.5% of the total variance and thus the remaining items of the empathy scale were discarded during the linear regression analysis. Based on the contents of this scale and the magnitude of the eigenvalue the item used during this analysis was "making clients to feel at ease".

#### Patient Enablement Instrument (PEI)

In the PEI patients were asked to rate six questions whether, as a result of their most recent visit, they felt to be able to cope with life, understand their illness, cope with their illness, keep healthy, confident about their health, and help themselves. All items were stated positively, each capturing responses via an ordinal scale. The scoring system was same or less = 0, better or more = 1 and much better or much more = 2. Taken together, the six items yield a score range of 0-12 [21]. The scale was reliable with Cronbach's alpha of 0.897. The items of the scale were subjected to factor analysis to identify the underlying components of the PEI. Only one factor with eigenvalue greater than one was produced. This factor accounted for 68.6% of the total variance. Therefore, the item of "able to cope with life" was considered to be a core ingredient of this scale and was used in subsequent analysis.

#### Perceived technical competency

Perceived technical competency of the provider is the subjective judgment of the patients about the professional skills and abilities of the health care provider to detect and manage their problem. It was measured by 8 items. Each item was scored on a five-point Likert scale ranging from strongly disagree (1) to strongly agree (5) which yields a score range of 8-40. The scale has high internal consistency (Cronbach's α = 0.910). The items cover physical examination, procedural steps to arrive at what is wrong, experience of the provider, etc. The items of the scale were subjected to factor analysis to look into the underlying components. Accordingly, one component with eigenvalue greater than one was identified and it accounts for 72.6% of the overall variance. This item was the one considering the conduct of "thorough physical examination by the provider". This was considered as the central constituent of perceived technical competency and the remaining items with eigenvalues less than one were discarded.

#### Perceived non-verbal communication of the provider

Non-verbal communication involves a range of communication activities of the providers that do not have linguistic contents. Perception of patients about the health care provider's non-verbal communication was measured by five items on a five-point Likert scale ranging from poor (1) to excellent (5). The items cover different aspects of non-verbal communication including making eye contact, forward leaning, open posture, facial expression, head nodding, hand gesture, emotional expressive and concerned voice tone. Reliability check showed that the scale has high internal consistency (Cronbach's α = 0.935). During factor analysis the scale was reduced to one item ("making eye contact") with eigenvalue of greater than one. This item explained 69.0% of the overall variance.

#### Actual consultation length

is the amount of time the patients spend with the health care provider in the consultation room. The consultation duration was recorded by the data collectors who measured the minutes elapsed between entry to and exit from the examination room.

#### Information sharing about illness

Five items with yes/no response were used to measure the extent to which relevant information was given to patients in relation to their illness. These items checked whether the patients were told the name and cause of their illness, to return if illness gets worse and how to prevent reoccurrence.

#### Patient satisfaction

Patient satisfaction with the latest visit was assessed using five items on a five-point Likert scale ranging from strongly disagree (1) to strongly agree (5). This scale was found to have high internal consistency (Cronbach's α = 0.887). The items in this scale include: "I am totally satisfied with the visit", "Something about my consultation is better", "I am not completely satisfied with my visit", "I would come back to this provider" and "I would send my friends or relatives to this provider". However, when factor analysis was computed, only one factor with eigenvalue greater than one was identified. This item ("I am totally satisfied with the visit") explained 69.6% of the overall variance and was used during further analysis.

Finally, all of these tools were translated into Afan Oromo (the local language) and back translated into English to check its consistency by different persons and the one in Afan Oromo was pre-tested on 5% of the sample size taken from a similar population before the start of the actual data collection. Data were collected by trained individuals who were not health professionals.

### Statistical analysis

The data were analyzed using SPSS statistical software version 16.0. The mean score of the scales was computed for patient enablement, perceived empathy, technical competency and non-communication. Each scale was subjected to factor analysis to investigate the underlying components and to reduce the number of items based on eigenvalue. Factors with eigenvalue less than one were discarded and only those with eigenvalue greater than one were considered in subsequent analysis. Factor score was computed for the item identified to represent the satisfaction scale by varimax rotation method. Using this regression factor score, multivariate linear regression analysis was performed and the effect of independent variables on the regression factor score of the dependent variable was quantified. In the first model, the effects of socio-demographic variables were assessed while in the second model the effects of institutional variables were considered. In the third model, variables related to interpersonal interaction were included. Finally, explanatory variables which had statistically significant association with the dependent variable (P < 0.05) were entered to the final regression model.

### Ethical consideration

The ethical issues of this study were reviewed and approved by the Ethical Committee of Jimma University. During the study, verbal informed consent was sought from all the respondents before the start of each interview.

## Results

### Socio-demographic characteristics of the respondents

Seven hundred sixty eight patients aged 15 years or older were interviewed yielding a response rate of 100%. Four hundred one (52.2%) of the interviewed patients were females. The mean age of the patients was 29.5 ± 10.6 years. More than six in ten (62.2%) of the respondents reside in the rural area. Four hundred twenty six (55.5%) of the respondents were married while 296 (38.5%) were single. Concerning educational status, 240 (31.3%) of the respondents cannot read and write and 147 (19.1%) of them have attended primary education (grade 1-6). Occupationally, 286 (37.3%) of the respondents were farmers.

### Socio-demographic predictors of patient satisfaction

The relationship between socio-demographic variables and satisfaction factor score is quantified in table 1 below. Socio-demographic variables were found to explain only 3.9% of the variability in the satisfaction factor score. Accordingly, marital status, residence, educational status and occupational status appeared to be statistically associated with satisfaction factor score. The satisfaction score for single respondents was decreased by an average of 0.314 (95%CI: -0.517 to -0.112) as compared to their married counterparts. Urban residents had 0.261 unit greater satisfaction score when compared to those from the rural area (95%CI: 0.090 to 0.431).

**Table 1:** Socio-demographic determinants of patient satisfaction with health care provider interaction at public health centres, central Ethiopia, January 2009

Socio-demographic Variables	No. (%)	p-value	Unstandardized B coefficient	95% CI for B
**Sex**				
Male	367 47.8)	.002	-.314	(-.517, -.112)
Female*	401 52.2)			
**Age**		.427	-.004	(-.013, .005)
**Residence**				
Urban	290 (37.8)	.003	.261	(.090, .431)
Rural*	478 (62.2)			
**Ethnicity**				
Oromo*	693 (90.3)			
Ahmara	57 (7.4)	.527	-.089	(-.366, .187)
Others	18 (2.3)	.155	-.352	(-.837, .133)
**Religion**				
Orthodox*	429 (55.9)			
Protestant	300 (40.1)	.162	-.105	(-.253, .043)
Others	39 (5.0)	.919	.018	(-.328, .364)
**Marital status**				
Single	296 (38.5)	.002	-.314	(-.517, -.112)
Married*	426 (55.5)			
Divorced	24 (3.1)	.233	-.264	(-.698, .170)
Widowed	22 (2.9)	.626	-.108	(-.545, .328)
**Educational status**				
Cannot read and write*	240 (31.3)			
Can read and write	123 (16.0)	.504	.077	(-.149, .303)
Grade 1-6	147 (19.1)	.175	.155	(-.069, .380)
Grade 7-12	207 (27.0)	.023	.282	(.038, .525)
Diploma and above	51 (6.6)	.233	.271	(-.175, .717)
**Occupational status**				
Farmer*	286 (37.3)			
House wife	158 (20.6)	.042	.227	(.008, .446)
Student	153 (19.9)	.389	.126	(-.162, .414)
Government employment	67 (8.7)	.923	.019	(-.375, .414)
Merchant	57 (7.4)	.387	-.141	(-.461, .179)
Others	47 (6.1)	.987	.003	(-.325, .330)

*References category (categories with highest frequency taken as reference categories)

### Institutional aspects and pattern of visit

In this survey the mean time taken by the respondents to reach the health centers, regardless of the means, was 82.4 minutes. Of the total respondents, 683 (88.9%) were new patients while the remaining were repeat visitors. More than nine in ten (96.2%) of the patients reported that they were interviewed in the language they understand. More than half (53.4%) of the patients reported that they were seen by a male health care provider while 496 (64.6%) of the respondents didn't previously know the health care provider who treated them. One hundred seventy six (22.9%) of the respondents claimed that their privacy was not respected during consultation. Moreover, 153 (19.9%) of the respondents felt that the consultation rooms did not provide adequate privacy. It was also found that 188 (24.5%) of the respondents did not tell all of their private issues related to their health condition to the health care provider. However, 747 (97.3%) and 676 (88.0%) of the respondents felt that the waiting areas and seats were comfortable, respectively.

### Institutional aspects and pattern of visit as predictors of patient satisfaction

Variables related to institutional aspects were entered into the second model and their relative effect and importance is presented in table 2. This model explained 15.4% of the variation in satisfaction among patients. Knowing the provider, frequency of visit, privacy of the room, feeling of privacy during consultation and telling one's own private issues had statistically significant association with patient satisfaction. Patients who knew the health care provider very well had an average increase of 0.499 unit in their satisfaction with their interaction with the provider compared to those who did not know the provider at all (95%CI: 0.129 to 0.870). Clients who did not tell their private issues had an average of 0.598 decrease in satisfaction score as compared to those who told their private issues to the provider (95%CI: -0.761 to -0.435). Moreover, patients who felt that they did not have privacy during consultation had an average decline of 0.400 in their satisfaction score as compared to those who felt there was sufficient privacy (95%CI: -0.570, -0.230).

**Table 2:** Institutional aspects and patient satisfaction with health care provider interaction at public health centres, central Ethiopia, January 2009

Institutional Variables	No. (%)	p-value	Unstandardized B coefficient	95% CI for B
**Sex of provider**				
Male*	410 (53.4)			
Female	358 (46.6)	.786	-.019	(-.158, .120)
**Knowing Health care provider**				
Know very well	35 (4.6)	.008	.499	(.129, .870)
Know well	57 (7.4)	.001	.488	(.205, .772)
Know little bit	180 (23.4)	.083	.171	(-.022, .363)
Don't know at all*	496 (64.6)			
**Frequency of visit in 12 months**				
Once*	563 (73.3)			
Twice	165 (21.5)	.045	-.229	(-.454, -.005)
Three times	29 (3.8)	.583	.102	(-.262, .466)
≥4 times	11 (1.4)	.093	-.518	(-1.123, .086)
**Type of visit**				
New*	683 (89.9)			
Follow up	85 (11.1)	.038	.274	(.015, .532)
**Involvement of other**				
Yes	198 (25.8)	.855	.014	(-.138, .167)
No*	570 (74.2)			
**Told your private issues**				
Yes*	580 (75.5)			
No	188 (24.5)	.000	-.598	(-.761, -.435)
**Privacy during consultation**				
Yes*	592 (77.1)			
No	176 (22.9)	.000	-.400	(-.570, -.230)
**Room privacy**				
Yes*	615 (80.1)			
No	153 (19.9)	.923	.009	(-.170, .188)
**Interviewed in your language**				
Yes*	739 (96.2)			
No	29 (3.8)	.050	-.356	(-.713, .000)
**Comfortable seat**				
Yes*	676 (88.0)			
No	92 (12.0)	.869	-.020	(-.256, .216)
**Clean waiting area**				
Yes*	709 (92.3)			
No	59 (7.7)	.130	-.233	(-.534, .069)

*References category (categories with highest frequency taken as reference categories)

### Interaction with the health care providers

Perceived empathy was rated as good, very good or excellent by 73.3% of the respondents. Similarly, about 35% the respondents strongly disagreed/disagreed about the technical competency of the providers. Eighty two percent of the respondents rated the non-verbal communication by the provider as good, very good or excellent on the five-point Likert scale. Moreover, 52.7% and 34.1% of the respondents reported that the consultation has enabled them to be able to cope better and much better with life respectively. Of the total number of patients included in this study, 406 (52.9%) and 287 (37.4%) reported that they were told their illness and its causes, respectively. However, only 254 (33.3%) of the respondents were given advices on how to prevent the reoccurrence of their current illness and other similar conditions in the future. More surprisingly, only 347 (45.2%) of the patients were told to return if their symptoms get worse.

On the other hand, the present study documented that the mean duration of consultation was 6.26 ± 2.55 minutes (range = 2-20 minutes). In the light of this finding, the consultation duration was below the mean value for 447 (62.1%) of the patients. However, the mean expected consultation duration was 14.02 ± 6.73 minutes (range = 4-30). Of all the consultations considered in this study, 624 (81.3%) lasted for less than the duration patients expected while 101 (13.2%) of the consultations took more than expected. Patients over expected the consultation duration by an average of 9.92 ± 6.33 minutes and under expected it by an average of 2.51 ± 2.03 minutes.

### Perceived interaction with the health care provider as predictor of satisfaction

Table 3 shows the regression estimates for the model with interaction related variables and patient satisfaction score. Accordingly, perceived empathy, perceived technical competency, non-verbal communication, patient enablement, being told the name of their illness, expectation, perceived consultation length and duration of illness were significant predictors of satisfaction. The model explained 51.8% of the variations in patient satisfaction. Respondents whose perceived poor empathy by the provider had an average drop of 0.389 in their satisfaction score as compared to the patients who perceived good empathy (95%CI: -0.621 to -0.155). Besides, as perceived empathy gets better, its effect on satisfaction score becomes more positive. Non-verbal communication had similar effect as that of perceived empathy. Respondents who rated non-verbal communication of the provider as poor have an average decrease of 0.515 unit in their satisfaction as compared to those who rated it as good (95%CI: -0.985 to-0.046). However, patients who witnessed excellent non-verbal communication had an average increase of 0.512 unit in satisfaction score as compared to those who reported good non-verbal communication (95%CI: 0.227 to 0.797). Moreover, Patients who disagreed to the technical competency of the providers had a satisfaction score 0.346 unit lower than those who agreed (95%CI: -0.475 to -0.217).

**Table 3:** Interpersonal interaction variables as predictors of patient satisfaction at public health centres, Central Ethiopia, January 2009

Explanatory Variables	No. (%)	p-value	Unstandardized B coefficient	95% CI for B
Provider made you feel at ease**				
Poor	47 (6.1)	.001	-.389	(-.621, -.156)
Fair	158 (20.6)			
Good*	277 (36.1)			
Very good	240 (31.3)	.000	-.355	(-.511, -.199)
Excellent	46 (6.0)	.215	.048	(.002, .428)
Provider examined me thoroughly**				
Strongly disagree	47 (6.1)	.504	-.079	(-.312, .153)
Disagree	222 (28.9)	.000	-.346	(-.475, -.217)
Neither	81 (10.5)	.003	-.278	(-.096, -.459)
Agree*	371 (48.4)			
Strongly agree	47 (6.1)	.000	.424	(.199, .650)
Provider's direct eye contact**				
Poor	10 (1.3)	.032	-.515	(-.985, -.046)
Fair	128 (16.7)	.000	-.469	(-.625, -.314)
Good*	340 (44.3)			
Very good	257 (33.5)	.000	.245	(.118, .372)
Excellent	33 (4.2)	.000	.512	(.227, .797)
Able to cope with life**				
Same/less	101 (13.2)	.016	-.132	(-.240, -.024)
Better*	405(52.7)			
Much better	262 (34.1)			
**Provider told you the name of your illness**				
Yes	406 (52.9)	.000	.231	(.107, .354)
No*	362 (47.1)			
**Provider told you to return if it gets worse**				
Yes*	486 (63.3)			
No	282 (36.7)	.005	-.177	(-.300, -.053)
**Provider told cause of your illness**				
Yes	287 (37.4)	.922	.006	(-.123, .136)
No*	481 (62.6)			
**Provider told enough about your treatment**				
Yes*	576 (75.0)			
No	192 (25.0)	.996	.000	(-.134, .135)
**Provider told you ways of preventing future recurrence**				
Yes*	516 (67.2)			
No	252 (32.8)	.528	-.046	(-.187, .096)
**Match with your expectation**				
Very much*	330 (43.0)			
Certain	317 (41.3)	.431	.048	(-.072, .169)
Only few	106 (13.7)	.000	-.473	(-.671, -.275)
Not at all	15 (2.0)	.003	-.623	(-1.027, -.219)
**Duration of stay with the provider**				
Very long	10 (1.3)	.781	.064	(-.385, .512)
Long	89 (11.6)	.149	.123	(-.044, .290)
Fair*	328 (42.7)			
Short	283 (36.8)	.318	.060	(-.058, .179)
Very short	58 (7.6)	.001	-.356	(-.571, -.141)

**Data reduction during factor analysis identified these questions to be sufficient measures of perceived empathy, perceived technical competency, non-verbal communication, and patient enablement in that order.

*References category (categories with highest frequency taken as reference categories)

### Levels of patient satisfaction with the visit

The levels of satisfaction of the respondents with health care provider interactions are displayed in table 4. It was found that 76 (9.9%) and 405 (52.7%) of the respondents were highly and moderately satisfied, respectively.

**Table 4:** Level of patient satisfaction with health care provider interactions at public health centres, Central Ethiopia, January 2009

Level of satisfaction	No.	%
Highly satisfied	76	9.9
Moderately satisfied	405	52.7
Neither satisfied nor dissatisfied	80	10.4
Somewhat dissatisfied	182	23.7
Highly dissatisfied	25	3.3

### Predictors of patient satisfaction with health care provider interactions

Table 5 shows the regression estimates and the relative effect of each predictor variable for patient satisfaction with health care provider interaction. Only variables which had statistically significant association with patient satisfaction are displayed in the table. The final model explained 62.6% of the variation in patient satisfaction. As depicted in table 5, non-verbal communication, perceived empathy, perceived technical competency and the extent to which patient expectation was met were strong predictors of patient satisfaction. For instance, respondents who perceived poor empathy by the provider had an average decrease of 0.319 in their satisfaction score compared to those who perceived good empathy (95%CI: -0558 to -0.079). For patients who perceived fair empathy by the provider, its effect on their satisfaction score was nil (unstandardized B coefficient = 0). However, respondents who perceived excellent empathy has an average increase of 0.187 unit in satisfaction score as compared to patients who perceived good empathy (95%CI: -0.030 to 0.404).

**Table 5:** Predictors of patient satisfaction with heath care provider interactions at health centres, Central Ethiopia, January 2009

Explanatory Variables	No. (%)	p-value	Unstandardized B coefficient	Standardized B coefficient	95% CI for B
**Educational status**					
Cannot read and write*	240 (31.3)				
Can read and write	123 (16.0)	.684	-.033	-.012	(-.190, .125)
Grade 1-6	147 (19.1)	.042	.153	.060	(.005, .300)
Grade 7-12	207 (27.0)	.103	.126	.056	(-.026, .278)
Diploma and above	51 (6.6)				
**Occupational status**					
Farmer*	286 (37.3)				
House wife	158 (20.6)	.007	.198	.080	(.054, .341)
Student	153 (19.9)	.822	.022	.009	(-.169, .212)
Government employment	67 (8.7)	.608	-.053	-.015	(-.257, .150)
Merchant	57 (7.4)	.625	.055	.014	(-.165, .274)
Others	47 (6.1)	.735	.040	.010	(-.194, .274)
**Knowing Health care provider**					
Know very well	35 (4.6)	.121	.225	.047	(-.059, .509)
Know well	57 (7.4)	.095	.189	.050	(-.033, .411)
Know little bit	180 (23.4)	.044	.154	.065	(.004, .304)
Don't know at all*	496 (64.6)				
**Frequency of visit in 12 months**					
Once*	563 (73.3)				
Twice	165 (21.5)	.046	-.174	-.071	(-.345, -.003)
Three times	29 (3.8)	.954	.002	.008	(-.277, .293)
≥4 times	11 (1.4)	.025	-.532	-.063	(-.997, -.068)
**Type of visit**					
New*	683 (89.9)				
Follow up	85 (11.1)	.023	.230	.072	(.031, .429)
**Provider made you feel at ease**					
Poor	47 (6.1)	.009	-.319	-2.614	(-.558, -.079)
Fair	158 (20.6)				
Good*	277 (36.1)				
Very good	240 (31.3)	.000	-.351	-4.323	(-.511, -.192)
Excellent	46 (6.0)	.091	.187	1.693	(-.030, .404)
**Provider examined me thoroughly**					
Strongly disagree	47 (6.1)		-.059	-.014	(-.292, .173)
Disagree	222 (28.9)		-.343	-.156	(-.476, -.211)
Neither	81 (10.5)		-.285	-.088	(-.467, -.104)
Agree*	371 (48.4)				
Strongly agree	47 (6.1)		.416	.100	(.191, .640)
**Provider's direct eye contact**					
Poor	10 (1.3)	.016	-.595	-.068	(-1.078, -.112)
Fair	128 (16.7)	.000	-.503	-.188	(-.658, -.348)
Good*	340 (44.3)				
Very good	257 (33.5)	.001	.224	.106	(.097, .351)
Excellent	33 (4.2)	.000	.560	.114	(.275, .846)
**Able to cope with life**					
Same/less	101 (13.2)	.014	-.068	-.137	(-.246, -.028)
Better*	405(52.7)				
Much better	262 (34.1)				
**Provider told you the name of your illness**					
Yes	406 (52.9)	.000	.218	.109	(.106, .331)
No*	362 (47.1)				
**Provider told you to return if it gets worse**					
Yes*	486 (63.3)				
No	282 (36.7)	.000	-.208	-.100	(-.324, -.092)
**Expectation meet**					
Very much*	330 (43.0)				
Certain	317 (41.3)				
Only few	106 (13.7)	.000	-.492	-.170	(-.671, -.312)
Not at all	15 (2.0)	.001	-.659	-.091	(-1.053, -.265)

*References category (categories with highest frequency taken as reference categories)

Perceived technical competency also has similar effects on patient satisfaction. As it moved from strongly disagree to strongly agree, the regression estimates improve from negative to positive. Moreover, respondents who were indifferent about the technical competence of the provider had an average drop of 0.285 unit in satisfaction score as compared to respondents who agreed (95%CI: -0.467 to -0.104). Similarly, patients who witnessed poor non-verbal communication had an average decrease of 0.595 unit in satisfaction score compared to those who reported good non-verbal communication (95%CI: -1.078 to -0.112). However, reporting excellent non-verbal communication has an average increase of 0.560 in satisfaction score compared to good non-verbal communication (95%CI: 0.275 to 0.846).

A closer look at the explanatory variables of patient satisfaction in this study revealed that very good empathy (Beta = -4.323), fair non-verbal communication (Beta = -0.188), fewer expectation met (Beta = -0.169) and disagreement to technical competency (Beta = -0.156) had greater negative influence on patient satisfaction. On the other hand, excellent non-verbal communication (Beta = 0.114) and being told the name of their illness (0.109) had pronounced positive influence on patient satisfaction.

## Discussion

Empathy is crucial to the effective achievement of patient centeredness in that it encapsulates sensitivity to both the informational and emotional aspects of communication [1,14,23,24]. The present study found that 73.3% of the respondents rated the empathy of the health care providers as good, very good or excellent which is lower than those reported in the United Kingdom [25,26]. Patient enablement indicates the quality of consultation with no indication of the process going on during consultation. In this study, only 34.1% of the respondents reported that the consultation has enabled them to cope with life much better. Though this is better than the findings in the United Kingdom studies cited earlier it could possibly imply poor quality of consultation and patient enablement in the study population.

Health care providers usually feel pressured to see more patients in short time, leading to concerns. This was found to be true in this study. The mean consultation duration for the patients was 6.26 minutes whereas the mean expected consultation duration was 14.02 ± 6.73 minutes. Surprisingly, 81.3% of the consultations lasted for less than the mean expected consultation duration. The consultation duration in this study is shorter than those found in previous studies [25-28]. Furthermore, health care providers have an ethical duty to teach the patients about their illness and promotion of health in every opportunity and consultation is an ample opportunity to do so [29]. However, 47.1% of the patients were not told the name of their illness. To make things worse, 62.6% of the respondents reported that the cause of their illness was not explained to them. This finding is much lower than findings in other studies carried out elsewhere [27,30,31]. Hence, there were so many missed opportunities to practice health education and promotion activities.

Non-verbal communication is a subtle form of communication that takes place in the initial three seconds after meeting someone for the first time and can continue throughout the entire interaction. It has a great impact as that of verbal communication but can be more easily misinterpreted [4]. Thus, it is important for the health care provider to be aware of the non-verbal messages they convey to their patients. In the present study, non-verbal communication significantly influenced patient satisfaction. This finding was supported by previous findings elsewhere [32,33].

Moreover, this study showed that 62.6% of the respondents were satisfied with the consultation. This finding is quite comparable with other findings in Ethiopia [34,35]. Finally, findings in this study indicated that perceived empathy, perceived technical competency, non-verbal communications, patient enablement and information sharing about the patient's illness were the main predictor variables of patient satisfaction with health care provider interaction. Similar findings were observed in some other studies [32,33,36]. However, it has to be noted that the findings of this study might suffer from response bias due to the fact that facility based studies produce more positive responses by the patient. This may result in relatively short-lived "halo effect" whereby patients feel more satisfied immediately after their consultation than they do afterwards.

## Conclusion

In conclusion, perceived technical competency, perceived empathy, non-verbal communication, being told the name of illness, frequency and type of visit, knowing the provider and patient enablement were the main predictor variables of patient satisfaction in this study. This shows that interpersonal interaction which relies on verbal and non-verbal communication is crucial in improving patient satisfaction and should be given due attention by the health care providers. Furthermore, better demonstration of empathy, information sharing about the patient's illness and greater efforts to improve patient enablement could positively affect the perception of the patients about the provider's competency and consequently their satisfaction.

## Competing interests

The authors declare that they have no competing interests.

## Authors' contributions

ZB was involved in the conception, design, analysis, report writing and manuscript writing. TA and MW had been involved in the design, analysis and interpretation of the data, and report writing. In addition, MW was involved in manuscript review. SM assisted with the design and report writing. All authors have read and approved the final version of the manuscript.

## Pre-publication history

The pre-publication history for this paper can be accessed here:

http://www.biomedcentral.com/1472-6963/10/78/prepub
